# Exploring Different Roles of *StWRKY4* and *StWRKY56* in Transgenic Potato Against Salt Stress

**DOI:** 10.3390/life15091389

**Published:** 2025-09-01

**Authors:** Nadia Gul, Sofia Baig, Xiaoliang Shan, Irum Shahzadi, Maria Siddique, Hongwei Zhao, Raza Ahmad, Jamshaid Hussain, Samina Khalid, Ayesha Baig

**Affiliations:** 1 Department of Biotechnology, COMSATS University Islamabad, Abbottabad Campus, Abbottabad 22044, Pakistan; nadiagul691@gmail.com (N.G.); irumayaz@cuiatd.edu.pk (I.S.); chishti@cuiatd.edu.pk (R.A.); jamshaidhussain@cuiatd.edu.pk (J.H.); saminakhalid@cuiatd.edu.pk (S.K.); 2 Independent Researcher, Abbottabad 22010, Pakistan; sofiabaig@live.com; 3 Department of Plant Pathology, College of Plant Protection, Nanjing Agricultural University, Nanjing 210095, China; sdausxl@126.com; 4 Department of Environmental Sciences, COMSATS University Islamabad, Abbottabad Campus, Abbottabad 22044, Pakistan; maria@cuiatd.edu.pk

**Keywords:** potato, WRKY, salt stress, RNAi, transgenic

## Abstract

WRKY transcription factors play an important role in transcriptional reprogramming associated with plant abiotic stress responses. In this study, the role of *Solanum tuberosum* (*S. tuberosum*; *St*) WRKY transcription factors *StWRKY4* and *StWRKY56* were explored in response to salt stress by generating transgenic potato lines using RNAi. The results showed that the total chlorophyll content in transgenic *StWRKY4* was 6.1 mg/g at 200 mM after 35 days; however, in *StWRKY56*, an elevated 12.6 mg/g total chlorophyll was observed which indicated different operating mechanisms of these *St*WRKY transcription factors under salt stress. Proline content increased to 1.0 mg/g in *StWRKY4* while it decreased to 0.54 mg/g in *StWRKY56* as compared to their respective control plants after 35 days at 200 mM of salt stress. For Na^+^/K^+^ ratios, *StWRKY4* and *StWRKY56* showed 32.3 and 5.5 values, respectively, in silenced plants under similar conditions. This shows contrasting trends in *StWRKY4* and *StWRKY56* for Na^+^/K^+^. However, the expression analyses of *StSOS1*s were found to be upregulated, whereas for *StNHX3*s these were found to be downregulated in *StWRKY4* and *StWRKY56* under salt stress. Thus, this study, for the first time, demonstrated the different but critical roles of *StWRKY4* and *StWRKY56* for fine-regulating salt stress tolerance in complex signaling network of potato plant.

## 1. Introduction

Potato (*S. tuberosum*) is a significant agricultural commodity worldwide due to its nutritional content, profitable yield and high industrial value [1]. It is regarded as an immensely important food crop because of its potential to reduce hunger, having three growing seasons per year and constantly being under the influence of climate/environment effect. Biotic and abiotic stressors have a detrimental impact on potato crop yield [2,3]. Potato crops can be severely affected by various abiotic factors including salinity [3]. Salt is a highly toxic element and the greatest hurdle faced internationally today, posing a hazard to over 800 million hectares of agricultural land [4,5]. Nearly 1.4 billion acres of land throughout the globe is impacted by soil salinity [6]. Salinity affects plant metabolism, growth rate, architecture, gene regulation and many other important functions. It is predicted that potato yield may potentially suffer an overall 50% decrease by the year 2050, under the influence of salinization [4,5].

The elevated level of soluble salts in the soil moisture hinders plant growth. Sodium chloride (NaCl) is the primary salt that contributes to salinization and exhibits an adverse impact on plants than Ca^+^ salts [4]. For proper growth and development, plants need to regulate a balance among (Na^+^) input and potassium (K^+^) efflux into plant tissues through ion homeostasis. The canonical salt overly sensitive (SOS) pathway exists within potato plants which is critical in regulating salt stress. SOS1, SOS2 and SOS3 are all interconnected SOS components that eliminate excessive accumulation of Na^+^ ions in the cell cytosol [3]. Similarly to this, high-affinity potassium transporters (HKTs) that regulate K^+^/Na^+^ homeostasis and sodium/hydrogen antiporters (NHXs) that take the cytosol’s accumulating K^+^ or Na^+^ ions within a vacuole are vital for salt stress tolerance [7]. Whilst the function of these important genes has been elucidated and extensively characterized, the underlying transcriptional network is not yet completely characterized in potato plants.

Drought and salinity tolerance are extremely low in potato and the development of novel varieties is sluggish because of their complex genetic makeup and narrow germplasm [7]. There is an urgent need for the development of novel potato cultivars that are resistant to stress factors under current environment influence. Consequently, understanding the intricate underlying process of salt tolerance is essential for recognizing and producing salt-tolerant plants which enhance agriculture productivity [8]. To survive under salt stress, plants develop a range of balancing mechanisms such as optimal growth rate, osmotic adjustment and ion homeostasis [8,9]. Perhaps the most important and critical adjustment mechanism adopted by plants under stress conditions is the transcriptional regulation maintained by transcription factors (TFs) [10].

TFs are regulatory proteins that work with a specific gene sequence by triggering or inhibiting the expression or transcription of desired genes [10]. Various TFs families based on conserved DNA-binding domain have been recognized in plants [10,11]. TFs play a key role in a number of regulatory processes in abiotic stress tolerance. Majority include DNA-binding WRKY domain (WRKY) Dehydration-responsive element binding (DREB), based on conserved MYB domain (MYB), Ethylene Response Factor (ERF) and bZIP gene families [10]. One of the largest and significant TFs families is the WRKY family [11]. WRKY TFs regulate numerous downstream stress-related genes that result in biochemical and physiological modifications required for plant adaptation under stress conditions. The WRKY TFs are based on conserved domain of nearly invariant stretch WRKYGQK followed by unique zinc-finger structure of Cystine (C) and Histidine (H) residues [12]. In potato, 79 WRKY TFs have been identified based on WRKYGQK domain and zinc-finger structure [13]. A total of 13 members belong to group I with two WRKYGQK domains and characteristic zinc-finger CX4CX22HXH or CX4CX23HXH. Group II has 52 members with one WRKY domain and CX5CX23HXH zinc-finger structure. The third group has 14 members with one WRKY domain and CX7CX24HXC zinc-finger motif. WRKY proteins play an important role in plant stress response as they may associate with a specific W-box motif (TTGAC/T) in promoter regions [13]. Different studies have demonstrated a vital role played by numerous WRKY family members in abiotic stress tolerance including *S. tuberosum St*WRKY. *St*WRKY1 conferred improved tolerance to *Phytophthora infestans* (*P. infestans*) and water stress [14]. *St*WRKY2 showed a strong resistance to *P. infestans* and improved drought tolerance [15]. Other WRKY TFs such as *Fortunella crassifolia Fc*WRKY40 significantly regulated salt tolerance by using SOS pathway [15]. In *Arabidopsis thaliana* (*A. thaliana*) and *Chrysanthemum morifolium*, *Cm*WRKY17 significantly affected salt stress tolerance via modulating the expression of stress-sensitive genes [16]. *Triticum aestivum Ta*WRKY2, *Ta*WRKY93 and *Ta*WRKY24 raised drought and salinity tolerance by accumulating osmo-protectants and managing oxidative stress [17,18]. In addition to reducing oxidative stress, *Tamarix hispida Th*WRKY4 enhanced *Arabidopsis* growth under salt stress and inhibited chlorophyll degradation [19]. Salt stress tolerance in *Arabidopsis* was decreased by overexpression of *Zea mays Zm*WRKY17 [20]. Transgenic *Populus alba* × *P. glandulosa Pag*WRKY75 reduced expression through RNAi indicated that the transgenic poplar lines were more sensitive to salt and osmotic stresses compared to the wild type [21]. Moreover, transgenic *Arabidopsis* overexpressing *Glycine max GmWRKY17* exhibited increased osmotic tolerance and *Malus domestica Md*WRKY56 positively regulated drought stress tolerance in transgenic *Arabidopsis*, apple calli and plants [22,23]. These studies indicate that WRKY TFs play a critical role in plant stress responses.

Salt stress applied to chickpea (*Cicer arietinum*; *Cs*) showed upregulation of *CsWRKY4* and *CsWRKY56* against NaCl based on the transcriptome data [24]. Similarly, date palm (*Phoenix dactylifera*; *Pd*) genome wise expression analysis showed *PdWRKY4* and *PdWRKY56* role in salt stress [25]. A recent study by Jiang et al. in 2025 showed *StWRKY4* upregulated and *StWRKY56* downregulated in response to salt stress based on RNA-seq data [26]. Thus, although different WRKY TFs have been recognized for their roles in abiotic stress tolerance and expression data for potato *St*WRKYs against salinity showed *StWRKY4* and *StWRKY56* upregulation, functional characterization of potato *St*WRKY TFs against salt stress remains unknown. Given this, we examined the role of potato *StWRKY4* and *StWRKY56* in salt stress tolerance using RNAi. Different parameters including chlorophyll, proline and Na^+^/K^+^ ratio were evaluated by developing transgenic potato lines of *StWRKY4* and *StWRKY56*. Our data suggests that *StWRKY4* and *StWRKY56* are differentially expressed under salt stress and could play opposite roles in salt stress tolerance.

## 2. Methodology

### 2.1. Plant Material and Growth Conditions

Potato (cv. Desiree) certified tubers/plants from Hazara Agriculture Research Centre, Abbottabad, Pakistan were used in this study. Plant tissue culture work was performed at Tissue culture lab of COMSATS University, Abbottabad. Potato plants were grown on Murashige and Skoog (MS) media for 30 days that were used as an explant source [27]. Culture conditions were 25 ± 2 °C and 16 h photoperiod (32 mmole/m.s), 45–55% relative humidity which were maintained throughout our experiments. Later, 1-month-old transgenic potato plants were transferred to a hydroponic system under similar conditions to evaluate *StWRKY4* and *StWRKY56* for salt stress.

### 2.2. Cloning of a Unique Sequence of StWRKY4 and StWRKY56 into RNAi Vector for Transformation into Agrobacterium Tumefaciens

The data for potato *StWRKY4* and *StWRKY56* were retrieved from PGSC database http://solanaceae.plantbiology.msu.edu/pgsc_download.shtml (accessed on 22 February 2021) to pick unique sequences for generating transgenic RNAi lines. The distinctiveness of particular segments was verified using GenBank, National Center for Biotechnology Information (NCBI) http://www.ncbi.nlm.nih.gov/Genbank/ (accessed on 22 February 2021). Online service Primer 3 https://primer3.ut.ee (accessed on 27 February 2021) was used to generate primers for the selected sequences. Distinct DNA sequences of 312 and 327 base pairs for *StWRKY4* and *StWRKY56* respectively, were amplified using 2X Taq DNA polymerase and cloned into Gateway pDONR vector [28]. Positive *Escherichia coli* DH5α with pDONR *StWRKY4* and *StWRKY56* were confirmed on 50 mg/L kanamycin Luria–Bertani (LB) agar media and colony PCR. Confirmed PCR products were cloned into pk7GW1WG11 vector (https://vectorvault.vib.be/; accessed on 13 September 2021) and transferred through electroporation into *Agrobacterium tumefaciens* (*A. tumefaciens*) GV3101. LB agar plates with kanamycin 50 mg/L, spectinomycin 25 mg/L along with colony PCR were used for the selection of positive clones. The PCR products were confirmed by sequencing using gene-specific primers (Table 1).

### 2.3. Phylogenetic Tree Construction for StWRKY4 and StWRKY56

Comparative analyses of *StWRKY4* and *StWRKY56* protein sequences were conducted based on sequence homology using *Arabidopsis thaliana* (*At*; thale cress), *Solanum pennellii* (*S. pennellii*; *Sp*; wild tomato), *Solanum lycopersicum* (*S. lycopersicum*; *Sl*; tomato), *Lycium barbarum* (*L. barbarum*; *Lb*; Goji berry) and *Capsicum chinense* (*C. chinense*; *Cs*; Chinese pepper) BLASTp (version BLAST+ 2.17.0) from NCBI [29]. The WRKY accession numbers for all WRKYs used in the phylogenetic analysis were *StWRKY4* (XM_006350101.2), *StWRKY56* (XM_006343863.2), *Cc*WRKY56 (PHU28072.1), *Sl*WRKY56 (XP_004245563.1), *Sl*WRKY4 X1 (XP_004235494.1), *Sl*WRKY4 X2 (XP_069151062.1), *Sp*WRKY45 (XP_015085317.1), *Sp*WRKY4_X1 (XP_015070047.1), *Lb*WRKY4 X2 (XP_060187049.1), *Lb*WRKY (XP_060211036.1), *Lb*WRKY4 X1 (XP_060187048.1), *Sp*WRKY4 X2 (XP_015070048.1), *At*WRKY11 (AEE85928.1). Integrated tool PhyloSuite (version 1.2.3) was used to build the phylogenetic tree, which incorporates tools such as IQ-TREE2, MAFFT and Gblocks [30]. The sequences were aligned and trimmed using MAFFT (version 7.526) [31]. Maximum likelihood analyses were performed with IQ-TREE v2.2.0, using models selected by Partition Finder [32,33]. The Shimodaira–Hasegawa-like approximate likelihood-ratio test (SH-aLRT) was used for fast branch tests to support the ML tree [34]. The best-fit model, JTT+G4, was used for phylogenetic inference, and the resulting tree was visualized with FigTree http://tree.bio.ed.ac.uk/software/figtree (accessed on 14 March 2025).

### 2.4. Development of StWRKY4 and StWRKY56 Transgenic Lines

A single *A. tumefaciens* colony with *StWRKY4* or *StWRKY56* was cultured in a 2 mL LB broth medium with selected antibiotics. The bacterial culture was placed in shaking incubator at 250 rpm under 28 °C for 48 h. Then, 600 to 800 μL culture was transferred to 50 mL of LB broth and incubated for 24 h at 225 rpm shaking. After centrifugation, the pellet was reconstituted in MS liquid medium with 74 mM acetosyringone and bacterial density was adjusted at OD 600–800. *A. tumefaciens* mediated transformation of transgenic potato plants containing *StWRKY4* and *StWRKY56* was performed [35]. Potato explants grown on MS medium were shifted to an MS inoculation medium. Leaves were infected with *A. tumefaciens* infection medium with continuous shaking at 50 rpm for 20 min. Potato explants were cultured on a co-cultivation medium for two days before being placed on MS regeneration and selection media supplemented with cefotaxime (250 mg/L) and 50 mg/L of kanamycin, along with growth hormones BAP and IAA (1:1.5 mg/L). Potato explants were grown in the growth chamber until the appearance of roots and shoots [32]. These potato plants were grown till tuber formation in 1:1 sterilized sand and soil mix autoclaved at 121 °C at 15 psi, and confirmed transgenic plants (Supplementary Figures S1 and S2) were transferred to hydroponics for further experiments (Supplementary Figure S3).

### 2.5. Salt Stress Analysis

For salt stress analysis, transgenic and non-transgenic potato plants were constantly aerated in pots containing 1/3rd of Hoagland’s solution. Plants were adapted to full strength solution with 25% increments [36]. Potato plants were exposed to sodium chloride (NaCl) stress at 0, 100 and 200 mM for 35 days. Leaves from untreated and treated plants were taken after 0 h, 21 and 35 days for biochemical analyses. Each parameter was tested with at least 3–4 biological replicates. Non-transgenic control plants (*St*WT); non-transgenic control salt stressed plants at 100 and 200 mM NaCl (*St*WT at 100 mM and *St*WT at 200 mM); transgenic silenced control plants *StWRKY4* and *StWRKY56*; transgenic silenced salt stressed plants at 100 and 200 mM NaCl (*StWRKY56* at 100 and 200 mM; *StWRKY56* at 100 and 200 mM); these six different treatments for each *StWRKY4* and *StWRKY56* were evaluated for number of leaves, number of roots, shoot length (cm) and root length (cm) using ruler scale measurements after 21 and 35 days of salt stress with detailed experimental design (Supplementary Figure S4).

### 2.6. Pigment Analysis for StWRKY4 and StWRKY56

Pigment analysis protocol was followed with some modifications for quantification of carotenoid, chlorophyll-a, chlorophyll-b and total chlorophyll content [37]. A total of 0.1 g of fresh leaves from non-silenced and silenced treated plants with salt treatments was crushed with pestle mortar. The sample solution was suspended in 10 mL of 80% acetone and was left overnight in the dark. Later, the mixture was centrifuged at 4 °C for 10 min at 5000 rpm. The supernatant from each sample was collected and absorbance was determined using spectrophotometer for chlorophyll-a, chlorophyll-b and carotenoids at 645 nm, 663 nm and at 470 nm, respectively, using T80+ UV/Vis (PG Instruments Ltd., Lutterworth, UK) spectrophotometer [37].

### 2.7. Proline Content

The proline content was evaluated using the Bates et al. method with some modifications [38,39]. A total of 0.2 g of fresh leaves from potato plants were mashed in a 1 mL of sulfosalicylic acid (3% *w*/*v*) to make a final homogenate powder and centrifuge at 6000 rpm for 5 min. The supernatant was mixed with 1 mL of glacial acetic acid (GAA) and 1 mL of ninhydrin reagent and placed in a water bath for an hour at 92 °C. The reaction was terminated by 5 min ice treatment and later mixed with 2 mL toluene. Tubes were vortexed for 5 min and supernatant was taken for UV absorbance measurement at 520 nm by using toluene blank with UV-Vis spectrophotometer. L-proline was used as standard control, and the proline concentrations were calculated via standard curve and estimated using formula provided by [35].

### 2.8. Sodium and Potassium Ratio Determination

For determination of K^+^ and Na^+^ concentrations, 100 mg of fresh non-silenced plants and transgenic silenced *StWRKY4* and *StWRKY56* were heated in a furnace at 520 °C for 5 h to turn them into ash. A mixture of nitric acid and perchloric acid in 5:1 was added. The solutions were filtered through 0.2 mm Whatman filter paper and the volume was raised to 15 mL using distilled water. The amounts of Na^+^ and K^+^ were measured in filtered suspensions using Atomic absorption spectrometry (PerkinElmer AAnalyst 700; Waltham, MA, USA). Various concentrations of K^+^ and Na^+^ were generated by using NaCl and KCl dilutions. The standard curve was used to determine Na^+^ and K^+^ contents in plant leaves in mg/g of dry weight [40].

### 2.9. RNA Extraction Quantification and Complementary DNA (cDNA) Synthesis

About five-week-old potato silenced and non-silenced plants grown in half-strength Hoagland nutrient solution was treated with 0, 100 and 200 mM NaCl for 0 h, 21 and 35 days. Leaves samples were immediately frozen in liquid nitrogen and stored at −80 °C. CTAB protocol was used for the purpose of extracting RNA as reported with minor modifications [41]. Briefly, leaves were treated with CTAB and later with chloroform–isoamyl alcohol (24:1). The supernatant was treated with 10 M lithium chloride and RNA pellet was obtained in 70% ethanol. The pellet was suspended in 50 µL ddH_2_O. The quantity of cDNA was checked at wavelengths of 260 and 280 nm while ddH_2_O was utilized as a blank control by using Nano Drop spectrophotometer (Colibri Microvolume Spectrometer; Berthold Technologies GmbH & Co. KG, Bad Wildbad, Germany). WizScript High Capacity (cDNA synthesis kit; Wizbiosolutions Inc., Seongnam-si, Republic of Korea) was used to synthesize cDNA. The reaction mixture was formed using 10X reaction buffers 2 μL, 20X dNTPs mixture 1 μL, Random hexamer 2 μL, WizScript RTase 1 μL, RNase Inhibitor 0.5 μL and RNase free water 4 μL and 10 μL RNA. The reaction was left to incubate at 25 °C for about 10 min, 37 °C to 120 min and terminated at 85 °C for 5 min [41].

### 2.10. Quantitative Real-Time qPCR

The step-one software (Applied Biosystem; Foster city CA, USA) version 2.3 was used to carry out real-time PCR. The ROX qPCR Mastermix SYBR Green 2X (WizPure; Bioquest, Inc., Pleasanton, CA, USA) was used in 48-well plate. *StActin* was used as internal control for comparative data analysis. The reaction of control and silenced plants were carried out in duplicate. Then, 200 ng of cDNA as a template was added to qRT-PCR reaction mixture. The sequence of *StActin*, gene-specific primers and salt stress primers used in this study are mentioned in Table 1 [42,43]. Amplification was carried out using WizPure SYBR Green. The reaction mixture of 20 µL with 2 µL cDNA, 1 µL primer pair, 10 µL RT-qPCR Master Mix and 6 µL autoclaved deionized double-distilled H_2_O was prepared. The reaction had an initial denaturation step of 5 min at 95 °C, 40 cycles and had three steps: denaturation for 45 s at 95 °C, annealing for 45 s at 62 °C and extension for 1 min at 72 °C. The Ct values of different plants were analyzed statistically using step-one software 9 Applied Biosystems (version 2.3; Waltham, MA, USA). In each reaction run, the gene of interest and reference gene were tested. Relative fold expression was calculated by ΔΔCt method [44].

### 2.11. Statistical Analysis

The results from the research work were provided as means ± standard deviation for a minimum of three separate samples. R-program was used to examine quantitative data using two-way analysis of variance ANOVA at *p* ≤ 0.05 and the Duncan multiple range test (DMRT) [45].

## 3. Results

### 3.1. Development of Transgenic Potato StWRKY4 and StWRKY56

**Figure 1:**
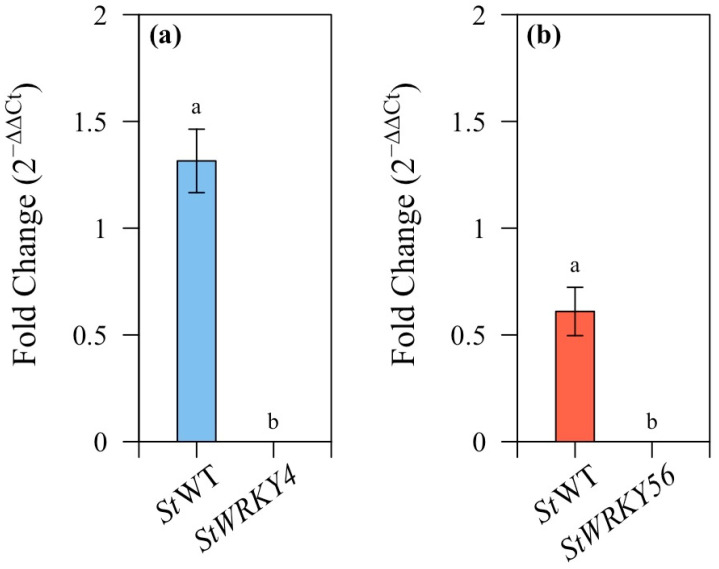
*StWRKY4* and *StWRKY4* expression analyses in transgenic and wild-type potato plants. (**a**) *St*WT:*StWRKY4* and (**b**) *St*WT:*StWRKY56*. Data are presented as relative fold change with mean values ±SD. Means with different letters were significantly different at *p* ≥ 0.05.

Transgenic *StWRKY4* and *StWRKY56* potato lines (Supplementary Figure S2) were used for salt stress analysis. RNAi-mediated gene silencing was used for generating transgenic *StWRKY4* and *StWRKY56* plants that were confirmed for downregulation through expression analysis as compared to control wild-type potato plants (Figure 1).

### 3.2. Phylogenetic Tree for StWRKY4 and StWRKY56

**Figure 2:**
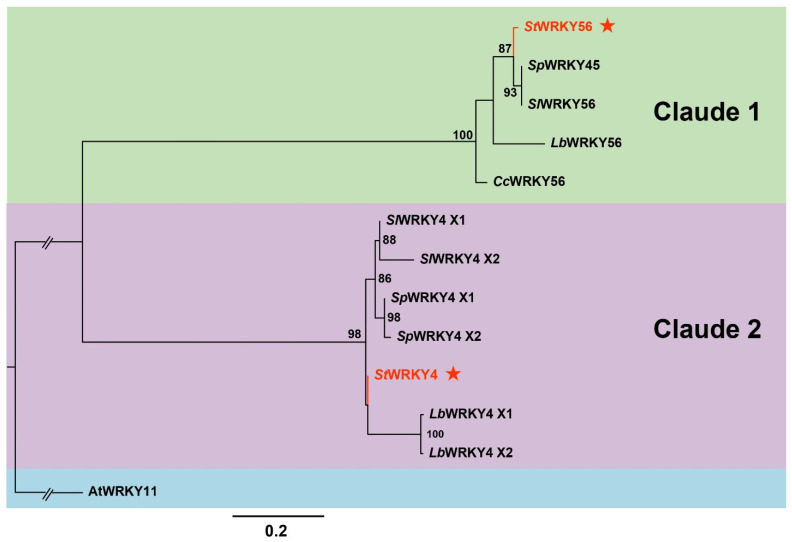
Phylogenetic tree based on WRKY protein sequences, illustrating the evolutionary relationships of *StWRKY4* and *StWRKY56 (*marked with red stars) along with *Arabidopsis thaliana* (thale cress), *Solanum pennellii* (wild tomato), *Solanum lycopersicum* (tomato), *Lycium barbarum* (Goji berry) and *Capsicum chinense* (Chinese pepper) with *At*WRKY11 used as an outgroup. The phylogenetic tree was inferred using IQ-TREE v2.2.0 with the maximum likelihood method. The tree robustness was assessed with 20,000 ultrafast bootstraps and the Shimodaira–Hasegawa-like approximate likelihood-ratio test.

A phylogenetic tree was constructed based on protein sequence homology of *StWRKY4* (XM_006350101) and *StWRKY56* (XM_006343863) along with 11 derived sequences similar to the two WRKY TFs. These homology sequences used were from *A. thaliana*, *S. pennellii*, *S. lycopersicum*, *L. barbarum* and *C. chinense*. *A. thaliana*, *At*WRKY11 was used as an outgroup in the phylogenetic tree (Figure 2). *StWRKY4* was found closely associated with *L. barbarum Lb*WRKY4 and *S. pennellii Sp*WRKY4 while *StWRKY56* was found related to *S. pennellii Sp*WRKY56 and *S. lycopersicum Sl*WRKY56.

### 3.3. Number of Leaves, Number of Roots, Shoot Length and Root Length

**Figure 3:**
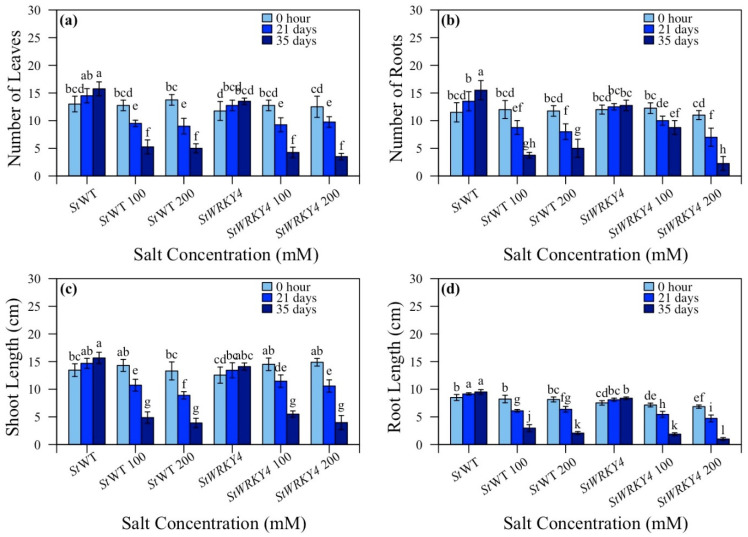
Effect of silencing of *StWRKY4* on plant growth in response to salt stress. (**a**) Number of leaves; (**b**) number of roots; (**c**) shoot length; and (**d**) root length in five-week-old *Solanum tuberosum* (*St*) wild-type (*St*WT), *St*WT at 100 and 200 mM, silenced *StWRKY4*, silenced *StWRKY4* at 100 and 200 mM were evaluated for salt stress. Data were observed after 0 h, 21 days and 35 days for each treatment. Duncan’s multiple range test was applied with different letters on bars and shows significantly different means at *p* < 0.05. Data are represented by means ± SD with three replicates.

*StWRKY4* transgenic potato plants were evaluated for number of leaves, number of roots, shoot length and root length against salt stress (Figure 3 and Supplementary Table S1). *StWRKY4* non-silenced plants/silenced plants showed number of leaves in ratios; 5.3:4.3 at 100 and 5.0:3.5 at 200 mM after 35 days, respectively (Figure 3a). For *StWRKY4*, in non-silenced/silenced plants the number of roots were 3.8:8.8 and 5.0:2.3 at 100 and 200 mM after 35 days (Figure 3b). Shoot length was 4.9:5.5 and 3.9:4.0 cm at 100 and 200 mM salt stress after 35 days (Figure 3c). Root length was 6.1:5.5 and 6.4:4.7 cm vs. 3.0:1.9 and 2.0:1.0 after 21 and 35 days at 100:200 mM salt stress for non- silenced wild-type/silenced *StWRKY4* plants (Figure 3d).

**Figure 4:**
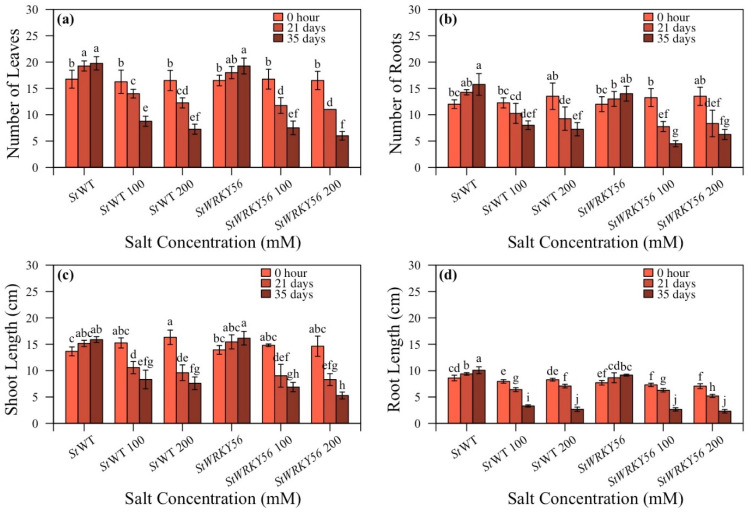
Effect of silencing of *StWRKY56* on plant growth in response to salt stress. (**a**) Number of leaves; (**b**) number of roots; (**c**) shoot length; and (**d**) root length of five-week-old *Solanum tuberosum* (*St*) wild-type (WT), WT with 100 and 200 mM, silenced *StWRKY56* and silenced *StWRKY56* with 100 and 200 mM were evaluated for salt stress. Data were recorded after 0 h, 21 days and 35 days for each treatment. Duncan’s multiple range test was applied with different letters on bars and shows significantly different means at *p* < 0.05. Data are represented by means ± SD with three replicates.

For *StWRKY56*, the number of leaves for non-silenced wild-type/silenced *StWRKY56* plants were 8.8:7.5 and 7.3:6.0 at 100 and 200 mM after 35 days (Figure 4a). The number of roots with non-silenced/silenced plants were 8.0:4.5 and 7.6:6.3 at 100 and 200 mM after 35 days (Figure 4b). For *StWRKY56*, it was noted that for non-silenced and silenced plants at 100:200 mM salt stress, shoot length was 8.3:6.9 and 7.6:5.3 cm after 35 days while root length was observed to be 3.3:2.7 and 2.7:2.3 cm with 100 and 200 mM salt stress at 35 days (Figure 4c,d). This indicated that silenced *StWRKY56* has reduced shoot length while *StWRKY4* has reduced root length as compared to non-silenced plants under salt stress.

### 3.4. Chlorophyll and Carotenoid Content

**Figure 5:**
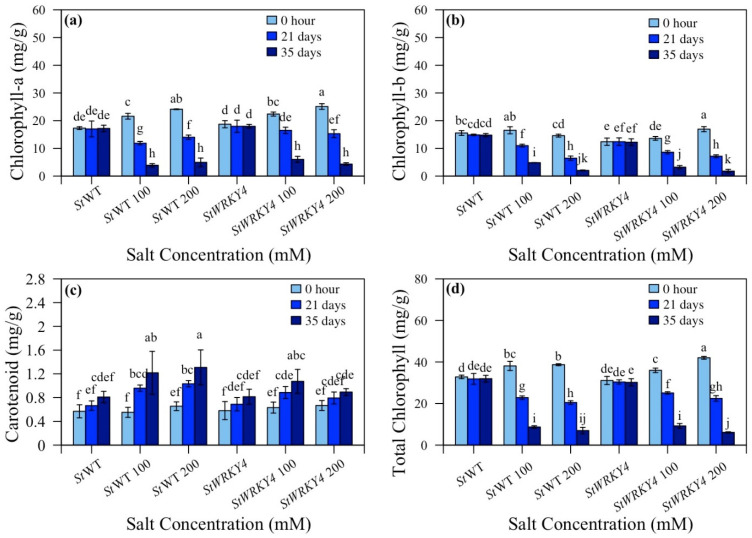
Effect of silencing of *StWRKY4* on plant pigments in response to salt stress. (**a**) Chlorophyll-a; (**b**) Chlorophyll-b; (**c**) Carotenoid; (**d**) Total chlorophyll in five weeks old *Solanum tuberosum* (*St*) wild-type (*St*WT), *St*WT at 100 and 200 mM, silenced *StWRKY4*, silenced *StWRKY4* at 100 and 200 mM were evaluated for salt stress. Data were recorded after 0 h, 21 days and 35 days for each treatment. Duncan’s multiple range test was applied with different letters on bars and shows significantly different means at *p* < 0.05. Data are represented by means ± SD with three replicates.

**Figure 6:**
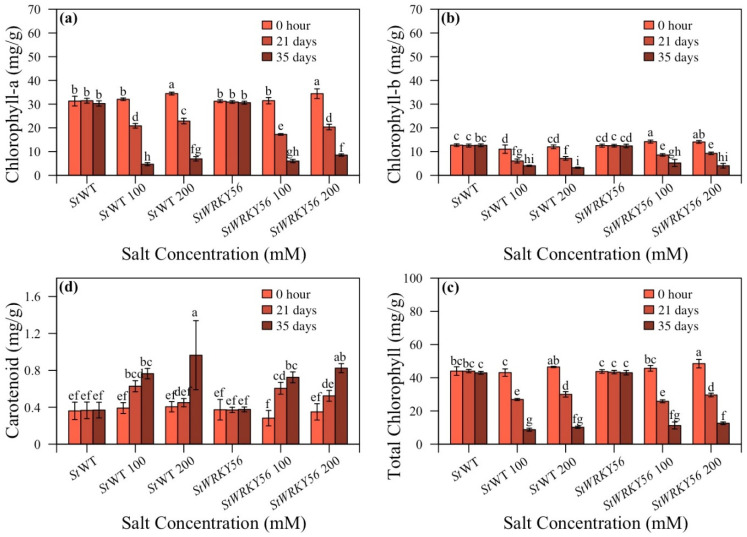
Effect of silencing of *StWRKY56* on plant pigments in response to salt stress. (**a**) Chlorophyll-a; (**b**) chlorophyll-b; (**c**) carotenoid; (**d**) total chlorophyll in five-week-old *Solanum tuberosum* (*St*) wild-type (*St*WT), *St*WT at 100 and 200 mM, silenced *StWRKY56*, silenced *StWRKY56* at 100 and 200 mM were evaluated for salt stress. Data were recorded after 0 h, 21 days and 35 days for each treatment. Duncan’s multiple range test was applied with different letters on bars and shows significantly different means at *p* < 0.05. Data are represented by means ± SD with three replicates.

Transgenic *StWRKY4* and *StWRKY56* potato plants were observed for chlorophyll and carotenoid content under salt stress (Figure 5 and Figure 6 and Supplementary Table S1). *StWRKY4* non-silenced plants/silenced plants showed 3.9:6.0 mg/g at 100 and 4.9:4.3 mg/g at 200 mM after 35 days and decreased chlorophyll-a content in silenced plants after 35 days with 200 mM salt stress (Figure 5a). Chlorophyll-b content was 4.8:3.2 mg/g and 2.0:1.7 mg/g in non-silenced/silenced *StWRKY4* plants at 100 and 200 mM while carotenoid content was 1.3:0.89 mg/g at 200 mM salt stress after 35 days (Figure 5b,c). Total chlorophyll content decreased for *StWRKY4* by 8.7:6.9 mg/g; 9.2:6.1 mg/g after 35 days at 100 and 200 mM salt stress for non- silenced/silenced plants (Figure 5d).

For *StWRKY56*, chlorophyll-a was 4.7:6.9 mg/g and 6.0:8.5 mg/g for non-silenced/silenced plants at 100 and 200 mM after 35 days (Figure 6a). For *StWRKY56*, chlorophyll-b was 3.2 and 4.0 mg/g for non-silenced/silenced plants at 200 mM; however, a lower trend was observed for carotenoids with 0.96:0.82 mg/g for non-silenced/silenced plants at 200 mM after 35 days (Figure 6b,c). For total chlorophyll in *StWRKY56*, non-silenced/silenced plants have 8.7:11.2 mg/g; 10.2:12.6 mg/g values at 100 and 200 mM salt stress after 35 days (Figure 6d). The data showed a somewhat decreased trend in carotenoid contents for both *StWRKY4* and *StWRKY56*; however, interestingly, silenced *StWRKY56* had increased chlorophyll content as compared to non-silenced plants under salt stress.

### 3.5. Proline Content and Na^+^/K^+^ Ratio

**Figure 7:**
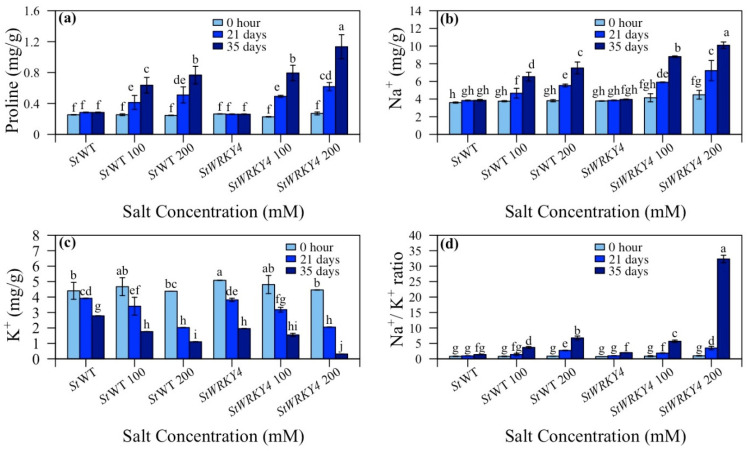
Silenced *StWRKY4* potato plants in response to salt stress. (**a**) Proline; (**b**) Na^+^ content; (**c**) K^+^ content; and (**d**) Na^+^/K^+^ ratio in five-week-old *Solanum tuberosum* (*St*) wild-type (*St*WT), *St*WT at 100 and 200 mM, silenced *StWRKY4*, silenced *StWRKY4* at 100 and 200 mM were evaluated for salt stress. Data were recorded after 0 h, 21 days and 35 days for each treatment. Duncan’s multiple range test was applied with different letters on bars and shows significantly different means at *p* < 0.05. Data are represented by means ± SD with three replicates.

**Figure 8:**
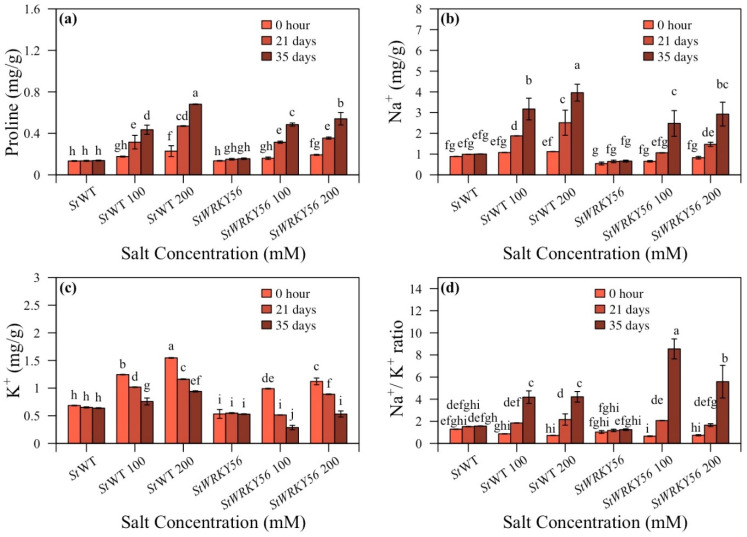
Silenced *StWRKY56* potato plants in response to salt stress. (**a**) Proline; (**b**) Na^+^ content; (**c**) K^+^ content; and (**d**) Na^+^/K^+^ ratio in five-week-old *Solanum tuberosum* (*St*) wild-type (*St*WT), *St*WT at 100 and 200 mM, silenced *StWRKY56*, silenced *StWRKY56* at 100 and 200 mM were evaluated for salt stress. Data were recorded after 0 h, 21 days and 35 days for each treatment. Duncan’s multiple range test was applied with different letters on bars and shows significantly different means at *p* < 0.05. Data are represented by means ± SD with three replicates.

Proline content is correlated with salt stress tolerance. It serves to reduce plant water potential thereby enhancing water uptake from the soil. It also helps decrease plant transpiration rate. Proline and Na^+^/K^+^ ratios were determined for *StWRKY4* and *StWRKY56* under salt stress (Figure 7 and Figure 8; Supplementary Table S1). It was observed that proline content was 0.6:0.8 mg/g in non-silenced/silenced plants at 100 mM and 0.8:1.1 mg/g in non-silenced/silenced plants at 200 mM after 35 days for *StWRKY4* (Figure 7a). Non-silenced/silenced plants had 4.6:5.9 and 5.5:7.2 mg/g (100 and 200 mM) and 6.5:8.8 and 7.5:10.1 mg/g (100 and 200 mM) of Na^+^ content at 21 and 35 days, respectively (Figure 7b). This shows that *StWRKY4* significantly accumulated Na^+^ ions as compared to non-silenced plants. In non-silenced/silenced plants, 1.8:1.6 and 1.1:0.3 mg/g (100 and 200 mM) of K^+^ ions content was observed after 35 days (Figure 7c). The Na^+^/K^+^ ratio was 1.4:1.9 and 2.7:3.5 (100 and 200 mM) and 3.7:5.7 and 6.8:32.3 (100 and 200 mM) in non-silenced/silenced plants after 21 and 35 days of salt stress. This shows increased Na^+^/K^+^ ratio for *StWRKY4* under salt stress (Figure 7d).

In case of *StWRKY56*, proline content in non-silenced/silenced plants was 0.3:0.5 and 0.7:0.5 mg/g (100 and 200 mM) salt stress after 35 days. This shows a decreased proline content at 200 mM at 35 days of salt stress for *StWRKY56* (Figure 8a). Na^+^ content was 3.2:2.5 and 3.9:2.9 mg/g (100 and 200 mM) while K^+^ content was 0.8:0.3 and 0.9:0.5 mg/g (100 and 200 mM) after 35 days of salt stress in non-silenced wild-type/silenced *StWRKY56* (Figure 8b,c). The Na^+^/K^+^ ratio in *StWRKY56* was 1.8:2.1 and 2.2:1.7 (100 and 200 mM) in non-silenced/silenced plants after 21 days whereas it was 4.2:8.5 and 4.2:5.6 (100 and 200 mM) in non-silenced/silenced plants after 35 days of salt stress. This shows that after 35 days, Na^+^/K^+^ ratio was significantly different than wild-type but less significant than *StWRKY56* after 35 days of salt stress (Figure 8d).

### 3.6. Quantitative Analysis of StSOS1 and StNHX3

**Figure 9:**
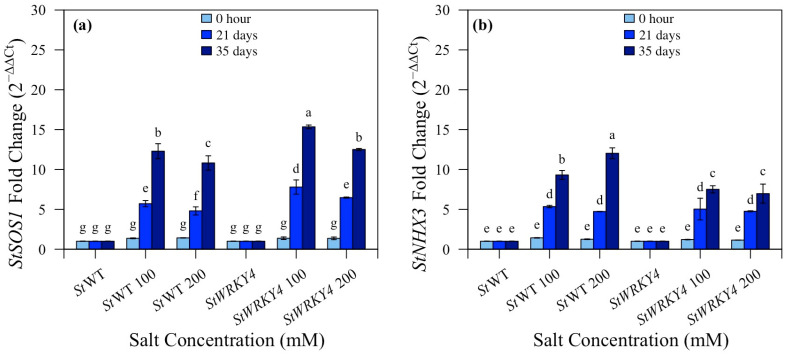
Expression analyses qRT-PCR of silenced *StWRKY4* potato plants. (**a**) *StSOS1* and (**b**) *StNHX3*. Five-week-old potato plants were treated with 100 and 200 mM salt concentrations. Leaves of control, treated control, silenced and treated silenced potato plants were harvested at 0 h, 21 and 35 days. Transcripts showed fold change after normalization with the internal control *StActin*. Significance letters of transcript fold change relative to control treatments with means ± SD with two replicates for each treatment were determined by Duncan’s multiple range test at *p* < 0.05.

Real-time qRT-PCR was performed for the expression analyses of *StSOS1* and *StNHX1* in silenced *StWRKY4* potato plants under 100 and 200 mM salt stress after 0 h, 21 and 35 days (Figure 9 and Supplementary Table S1). The expression level of *StSOS1* was calculated as fold increase 2^−ΔΔCt^ with 12.3:15.3; 10.8:12.5 (100:200 mM) after 35 days for *StWRKY4* in non-silenced vs. silenced plants. *StNHX1* in *StWRKY4* after 35 days was 9.7:7.5; 12.0:6.9 (100:200 mM) in non-silenced vs. transgenic silenced potato plants. This shows that the expression of *StSOS1*was upregulated in silenced *StWRKY4* whereas the expression of *StNHX1*was downregulated in silenced plants after 100 and 200 mM salt stress as compared to silenced control potato plants after 35 days.

**Figure 10:**
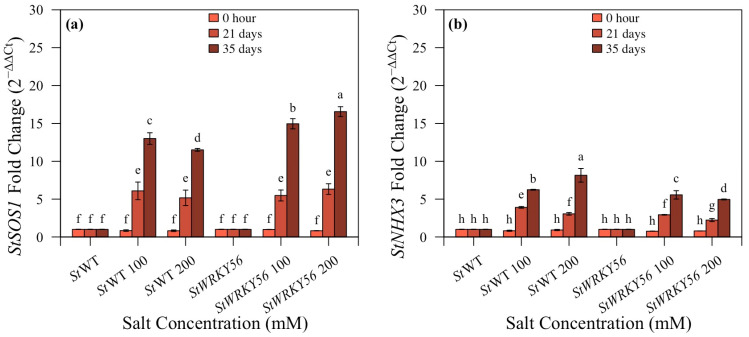
Expression analyses qRT-PCR of silenced *StWRKY56* potato plants. (**a**) *StSOS1* and (**b**) *StNHX3*. Five-week-old potato plants were treated with 100 and 200 mM salt concentrations. Leaves of control, treated control, silenced and treated silenced potato plants were harvested at 0 h, 21 and 35 days. Transcripts showed fold change after normalization with the internal control *StActin*. Significance letters of transcript fold change relative to control treatments with means ± SD with two replicates for each treatment were determined by Duncan’s multiple range test at *p* < 0.05.

It was observed that *StSOS1* in *StWRKY56* showed fold change of 13:15; 11.5:16.5 (100:200 mM) after 31 days salt stress, whereas *StNHX1* in *StWRKY56* showed a fold change of 3.9:2.9; 3.1:2.2 (100:200 mM) after 21 days and 6.2:5.5; 8.1:4.9 (100:200 mM) after 35 days in non-silenced and silenced transgenic plants under salt stress (Figure 10). This shows that the expression of *StSOS1* was significantly upregulated in silenced salt stressed plants, however the expression of *StNHX1* was downregulated (Figure 9 and Figure 10).

## 4. Discussion

WRKY TFs family has been extensively investigated due to its numerous roles in plant development and various stress responses [46,47]. WRKY TFs regulate multiple biological functions and modify gene expressions by a combination of positive and negative regulation [48,49]. In the current study, *StWRKY4* and *StWRKY56* were evaluated for their salt stress responses in potato plant. Unique exon sequences of *StWRKY4* and *StWRKY56* from the potato WRKY family were used to create transgenic RNAi potato plants of Desiree variety (Figure 1). A phylogenetic tree based on *StWRKY4* and *StWRKY56* depicted the clustering of these WRKYs into two evolutionary claudes 1 and 2 [49]. *StWRKY56* was closely grouped with tomato variants *S. pennellii* (*Sp*WRKY45), *S. lycopersicum* (*Sl*WRKY56) and distinctly with *L. barbarum* (*Lb*WRKY56), *C. chinense Cc*WRKY56. *StWRKY4* was relatively closely grouped with *Lb*WRKY4, *Sp*WRKY4 and distinctly with *Sl*WRKY4 (Figure 2).

In this study, silencing *StWRKY4* and *StWRKY56* had a differential effect on potato plants growth under salt stress. Based on morphological parameters, silenced *StWRKY4* had reduced number of roots and root length as compared to control plants than silenced *StWRKY56* under salt stress (Figure 3 and Figure 4). Overexpressed *Gossypium hirsutum GhWRKY34* in *Arabidopsis* had higher number of green cotyledons and root length in transgenic lines [50]. Similarly to this, Huang et al. in 2022 showed *OsWRKY54* CRISPR-Cas9 generated lines had reduced dry weight of root and shoot than wild-type plants under salt stress [51].

Our results showed that *StWRKY4* and *StWRKY56* silenced potato plants exhibited contrasting trends in chlorophyll content due to salt stress as compared to non-silenced plants (Figure 5 and Figure 6). *StWRKY4* results are comparable to the findings reported by Hichri et al. in 2017, which indicated an increased chlorophyll content in *SlWRKY3* overexpressed tomato plants in coping with salt stress [52]. *Dendranthema grandiflora DgWRKY5* overexpressed transgenic lines were also found to possess a higher rate of photosynthesis than WT [53]. Different chlorophyll contents in *StWRKY4* and *StWRKY56* under salt stress are similarly reported in *Abelmoschus esculentus Ae*WRKY32 and *Ae*WRKY70 overexpressed plants [54].

Carotenoids work like antioxidants, scavenging reactive oxygen species (ROS) within plants as well as safeguarding chlorophyll content. Salt stress triggers carotenoids reduction as well as cellular membrane disruption along with macromolecules failure [55,56]. In this study, carotenoids exhibited a significantly lower trend in silenced *StWRKY4* and *StWRKY56*. *StWRKY4* results are consistent with a report on silenced stressed *SlWRKY36* and *SlWRKY51* tomato plants that had considerably lower chlorophyll-a, carotenoid and total chlorophyll content than silenced plants [25].

An adaptive measure to salt stress is the proline response within the plant species, assisting not only in osmotic adjustment but also in ROS detoxification [57]. Proline helps in maintaining turgor pressure, mitigating salt stress while carrying out fundamental life-sustaining activities such as photosynthesis [58]. Our outcomes demonstrated that proline increased in silenced *StWRKY4* whereas it decreased in silenced *StWRKY56* at 100 mM and 200 mM NaCl stress after 21 and 35 days compared to non-silenced plants under similar conditions (Figure 7 and Figure 8). He et al. in 2024 showed in Okra plants similar outcomes with higher proline content in *AeWRKY70* and lower in *AeWRKY32* overexpressed plants [54]. Soybean *GmWRKY12* overexpressed in tomato plants and *SlWRKY81* silenced in tomato plants showed tolerance to salt and drought, respectively, with an increased proline content [59,60].

Intercellular Na^+^ ions management and regulation are vital plant strategies for sustaining ionic balance and growth performance under salt stress. It was indicated that Na^+^ content increased in *StWRKY4* and decreased in *StWRKY56* at 100 and 200 mM after 35 days as compared to non-silenced, wild-type plants under similar conditions. Our results also demonstrated increased Na^+^/K^+^ ratios in *StWRKY4* and *StWRKY56* at 200 mM after 35 days of salt stress as compared to their non-silenced control plants; however, Na^+^/K^+^ ratio in *StWRKY4* was significantly high (Figure 6 and Figure 7). *StWRKY4* trend is similar to higher sodium [Na^+^] and lower potassium [K^+^] in transgenic knockout/overexpressed *SlWRKY80* [60]. Thus, in contrast, total chlorophyll, proline and Na^+^ contents in silenced *StWRKY4* and *StWRKY56* show somewhat different mechanisms operating for these two WRKYs to alleviate salt stress.

In this study, *StSOS1* exhibited increased expression levels at 100 and 200 mM salt stress in *StWRKY56* and *StWRKY4* after 21 and 35 days as compared to their respective control non-silenced plants (Figure 8 and Figure 9). This indicated that the SOS system is the canonical essential regulatory node for ion homeostasis operating in potato plant. Liang et al. in 2023 showed *SOS1* used in this study increased expression under salt stress [42]. Conversely to *StSOS1s*, *StNHX3s* expression decreased in *StWRKY56* and *StWRKY4* under similar conditions (Figure 9 and Figure 10) [43]. Lower *HKT1*and *NHX4* means less Na^+^ in shoot and less sequestering in vacuole, respectively. Thus, in our study, *StSOS1s* and *StNHX3s* operated differentially under salt stress conditions.

In another study, *Medicago sativa Ms*WRKY33 TF rapidly accumulated under 200 mM salt stress after 2 weeks. The fresh biomass, dry weight and chlorophyll content of overexpressed *Ms*WRKY33 transgenic plants were significantly higher than those of control plants. *Ms*WRKY33 interacted with *Ms*CaMBP25 showing that it is involved in Ca^2+^ signal transduction. Additionally, *Ms*WRKY33 was also shown to directly bind to the promoter of *Ms*ERF5 and activating ROS scavenging pathway for high salt tolerance [61]. Similarly, it was found that WRKY45 in *Arabidopsis* played a vital role in salinity and osmotic stress by binding to W-box cis elements of responsive-to-desiccation *RD29A* promoter and activating ROS and stress-related genes expression [62]. Chen et al. in 2010 showed *Arabidopsis At*WRKY18 and *At*WRKY60 as positive regulators while *At*WRKY40 as negative regulator of ABA-induced abiotic stress [63]. It was found that *AtWRKY60* promoter W-boxes were recognized by *At*WRKY18 and *At*WRKY40 and both *At*WRKY18 and *At*WRKY40 activated *AtWRKY60* [63]. With ABA stress, *At*WRKY18 and *At*WRKY40 either as homodimers or heterodimers could bind to the W-boxes in *AtWRKY60* promoter, indicating an intricate relationship between negative and positive WRKY regulators in abiotic stress tolerance. Li et al. in 2025 recently indicated direct binding of rice *OsWRKY72* to Shoot K^+^ concentration 1 (SKC1), a *HKT1* promoter containing W-box regulating Na^+^/K^+^ ion homeostasis under salt stress [64]. Similarly, *Pyrus betulaefolia PbWRKY40* was found to bind the W-box element in the promoter region of the *Vacuolar-type-H^+^-ATPase* (*VHA-B*) and positively regulated salt stress tolerance [65]. Huang et al. in 2022 and Yu et al. in 2023 showed *OsWRKY54* and *OsWRKY53* directly binding to the promoter of *HKT1* in rice, respectively [51,66]. Based on this, it seems that WRKYs are involved in salt stress tolerance by directly binding to genes involved in ion homeostasis and ROS signaling/balancing. It therefore seems plausible that *St*WRKY3 and *StWRKY4*5 may bind directly to salt stress-responsive genes and work as homo- or heterodimers with other interacting *St*WRKYs for salt stress tolerance.

## 5. Conclusions

Our findings demonstrated significant roles of *StWRKY4* and *StWRKY56* in potato salt stress tolerance. *StWRKY4* and *StWRKY56* silenced transgenic lines showed similar trend in number of leaves, carotenoids, K^+^ and Na^+^/K^+^ ratios under salt stress. Shoot length, number of roots, root length, total chlorophyll, chlorophyll-a, Na^+^ and proline contents showed opposite roles in *StWRKY4* and *StWRKY56* at 200 mM NaCl after 35 days. Based on our results, it appears that *StWRKY4* and *StWRKY56* play opposite roles in salt stress tolerance in potato plants.

## Figures and Tables

**Table 1:** Primers used for *StWRKY4*, *StWRKY56*, *StNHX3*, *StSOS1* and *StActin*.

Primers	Forward Sequence	Reverse Sequence
*StWRKY4*	5′ GCTCCACCAACTCTACATTCCC 3′	5′ CAGAATGAGCAACAAGAGCCCC 3′
*StWRKY56*	5′ CCCTTGTGAAAAGCTAATGGAG 3′	5′ GCATGTGTGATGTGTACATCG 3′
*StNHX3*	5′ TTGGCACAGACGTGAACCTA 3′	5′ GTGGCTTCTGACCAGTGACA 3′
*St* *SOS1*	5′ TCCTGGAGACGGTAGCCAAA 3′	5′ ATTCCACCAATGGCAGCAGA 3′
*StActin*	5′ ATGAAGCTGTCCTTTTCACTTGTTTT 3′	5′ CTACATAGTATGCATGTCCGTATTT 3′

## Data Availability

The original contributions presented in this study are included in the article. Further inquiries can be directed to the corresponding authors.

## Supplementary Materials

The following supporting information can be downloaded at: https://www.mdpi.com/article/10.3390/life15091389/s1, Figure S1: PCR confirmation of (a) *StWRKY56* and (b) *StWRKY4* and *Agrobacterium tumefaciens* colonies (c) *StWRKY4* and (d) *StWRKY56*; Figure S2: (a,b) *StWRKY4* on MS selection media containing Cefotaxime, Kanamycin and BAP: IAA; (c) Lane 1 represents 100 bp DNA ladder; Lane 2 represents no band amplification with Kanamycin *NPTII* primers from wild type; Lane 3 represents PCR in transgenic *StWRKY4* with Kanamycin *NPTII* primers; (d,e) *StWRKY56* on MS selection media containing cefotaxime, Kanamycin and BAP: IAA; (f) Lane 1 represents no band amplification with Kanamycin *NPTII* primers from wild type; Lane 2 represents PCR in *StWRKY56* with Kanamycin *NPTII* primers; Lane 3 represents 100 bp DNA ladder; Figure S3: Wild type (*St*WT), transgenic plants *StWRKY4* and *StWRKY56* grown on MS media were transferred to constantly aerated pots with Hoagland solution in hydroponics. (a) *St*WT control plants, *StWRKY4* and *StWRKY56* transgenic lines in MS media; (b) *St*WT wild type plants at 0, 100 and 200 mM salt stress for 0 hour, 21 and 35 days; (c) *StWRKY4* under 0, 100 and 200 mM salt stress salt stress treatments for 0 hour, 21 and 35 days; (d) *StWRKY56* transgenic RNAi lines with 0, 100 and 200 mM salt stress treatment for 0 hour, 21 and 35 days. This assay was repeated and data was recorded based on treatment ± SD with *p* < 0.05 Duncan multiple range test; Figure S4: Wild type, transgenic *StWRKY4* and transgenic *StWRKY56* were grown for 1 month in the growth chamber in pots containing sterilized sand and soil mix in 1:1 ratio at 25 °C for 16 h photoperiod for 4 weeks and later shifted to hydroponic setup. After 35 days, ~27 wild type and ~27 transgenic plants each for *StWRKY4* and *StWRKY56* were treated with 0, 100, and 200 mm NaCl for further analysis at 0 hour, 21 and 35 days. All experiments were performed in biological triplicates; Table S1: Effect of salt stress on the growth, chlorophyll content, Proline, Na^+^/K^+^ ratio, *SOS1* and *NHX3* expressions of *StWRKY4* and *StWRKY56*. Different letters indicate a significant difference at *p* < 0.05 among six different treatments at three different time points according to the Duncans Multiple range test. Values are means ± SD.
