# Supplementary material for: Exploring Different Roles of StWRKY4 and StWRKY56 in Transgenic Potato Against Salt Stress
Source: Life (Basel). 2025 Sep 1;15(9):1389. doi: 10.3390/life15091389 (PMC12471359; doi:10.3390/life15091389)
Supplement: Supplementary file 1 [file life-15-01389-s001.zip › life-3817773-supplementary.pdf]

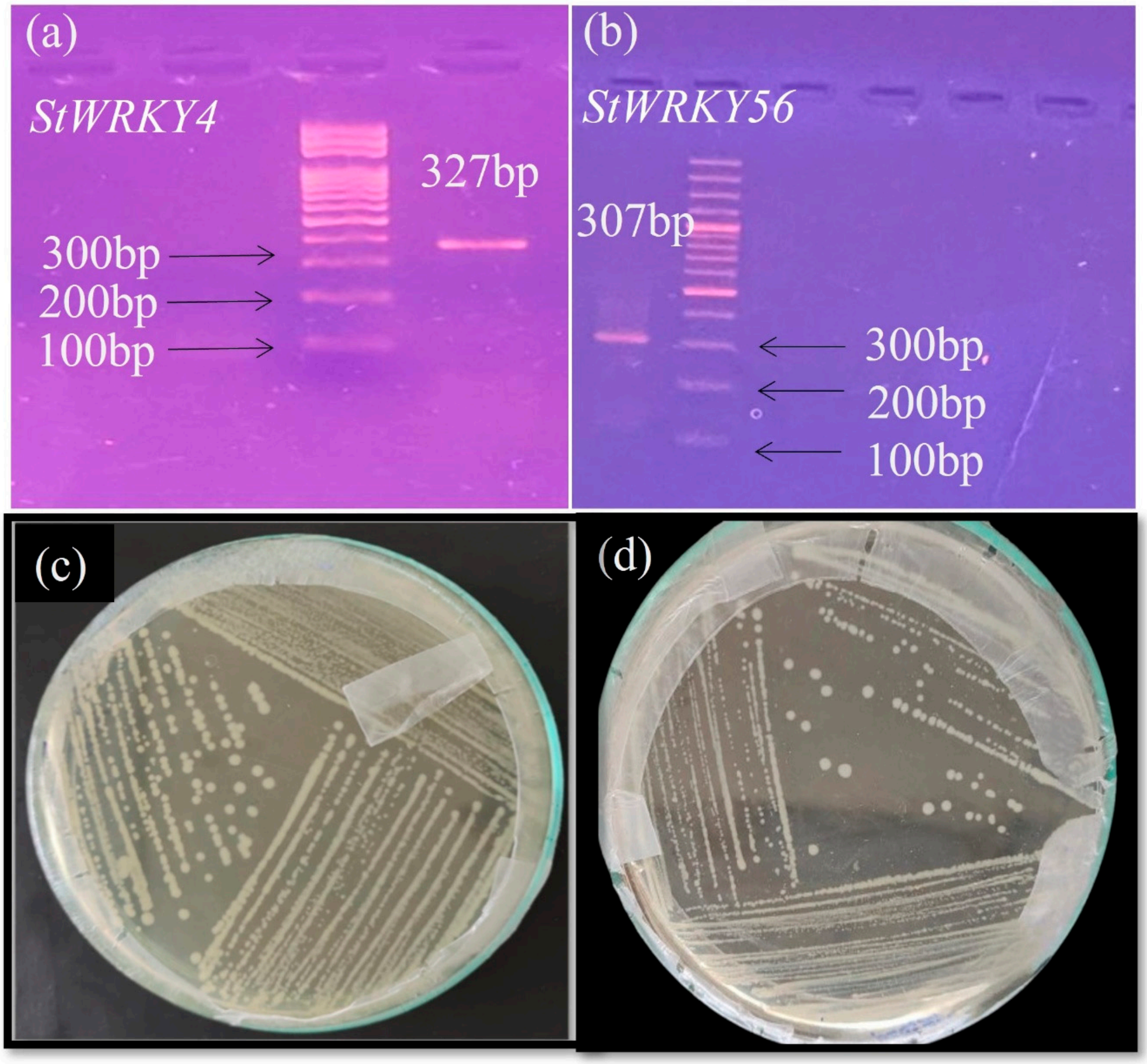

Supplementary Figure S1. PCR confirmation of (a) *StWRKY56* and (b) *StWRKY4* and *Agrobacterium tumefaciens* colonies (c) *StWRKY4* and (d) *StWRKY*56.

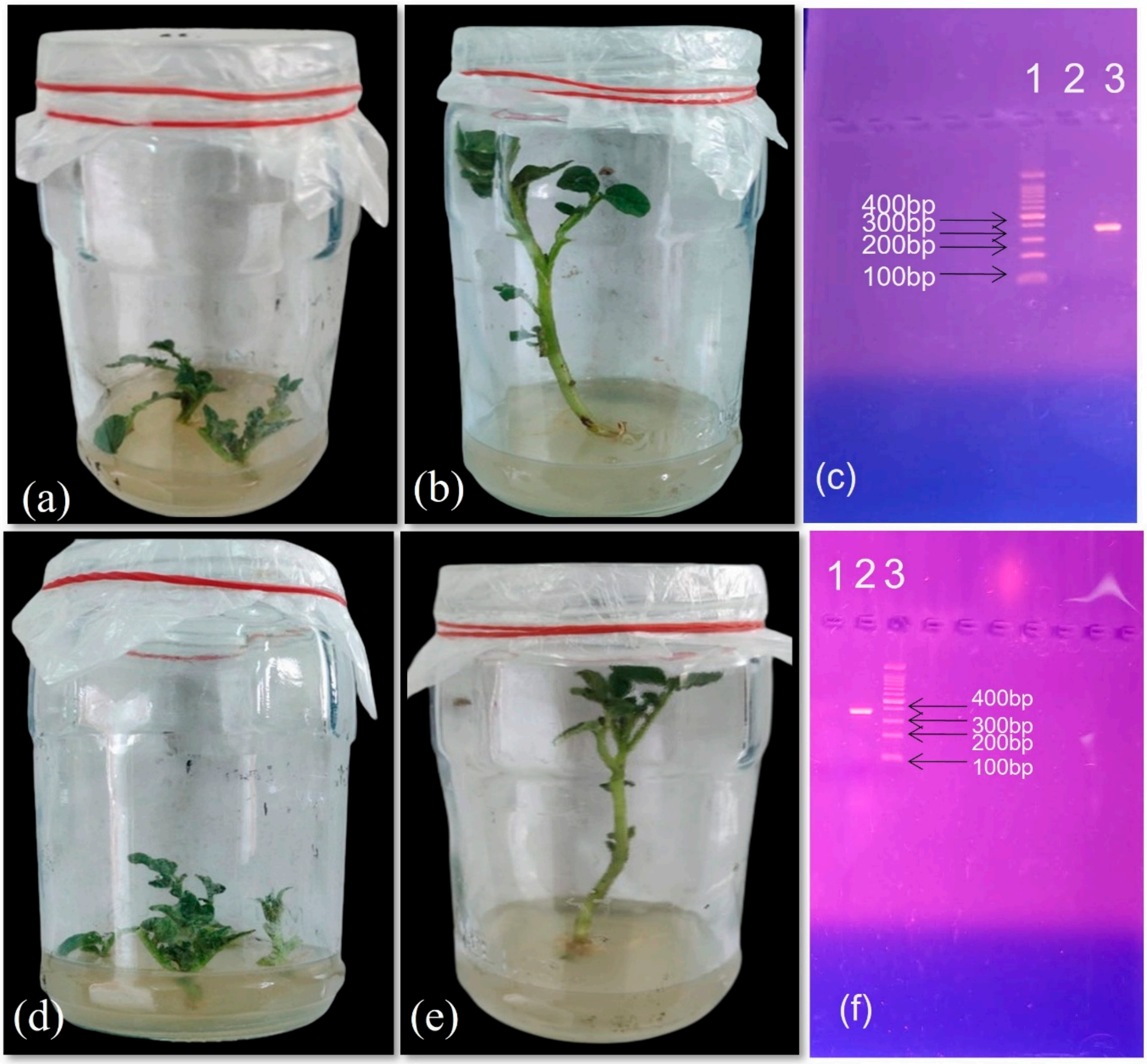

Supplementary Figure S2. (a-b) *StWRKY*4 on MS selection media containing Cefotaxime, Kanamycin and BAP: IAA; (c) Lane 1 represents 100 bp DNA ladder; Lane 2 represents no band amplification with Kanamycin *NPTII* primers from wild type; Lane 3 represents PCR in transgenic *StWRKY4* with Kanamycin *NPTII* primers; (d-e) *StWRKY56* on MS selection media containing cefotaxime, Kanamycin and BAP: IAA; (f) Lane 1 represents no band amplification with Kanamycin *NPTII* primers from wild type; Lane 2 represents PCR in *StWRKY56* with Kanamycin *NPTII* primers; Lane 3 represents 100 bp DNA ladder.

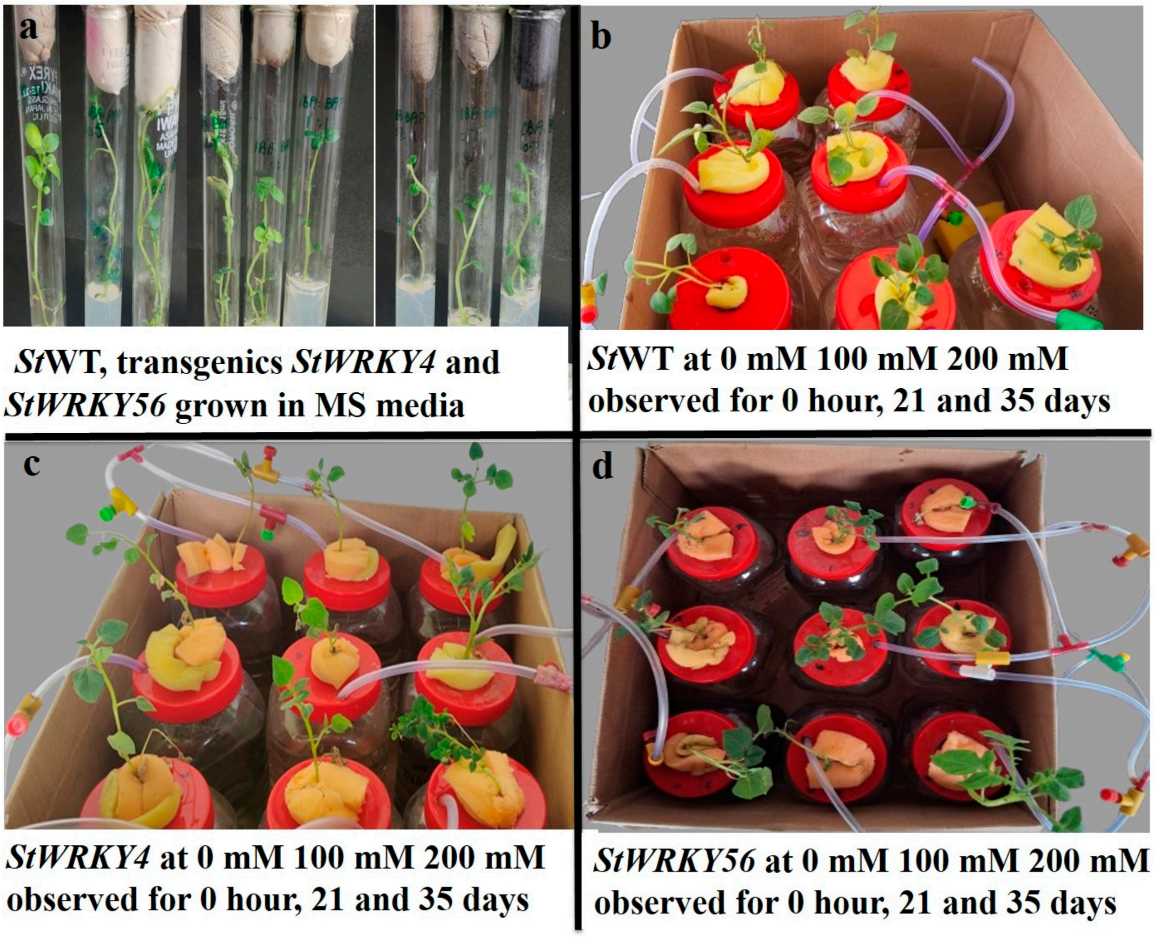

Supplementary Figure S3. Wild type (*St*WT), transgenic plants *StWRKY4* and *StWRKY56* grown on MS media were transferred to constantly aerated pots with Hoagland solution in hydroponics. (**a**) *St*WT control plants, *StWRKY4* and *StWRKY56* transgenic lines in MS media; (**b**) *St*WT wild type plants at 0, 100 and 200 mM salt stress for 0 hour, 21 and 35 days; (**c**) *StWRKY4* under 0, 100 and 200 mM salt stress salt stress treatments for 0 hour, 21 and 35 days; (**d**) *StWRKY56* transgenic RNAi lines with 0, 100 and 200 mM salt stress treatment for 0 hour, 21 and 35 days. This assay was repeated and data was recorded based on treatment $\pm$ SD with $P \leq 0.05$ Duncan multiple range test.

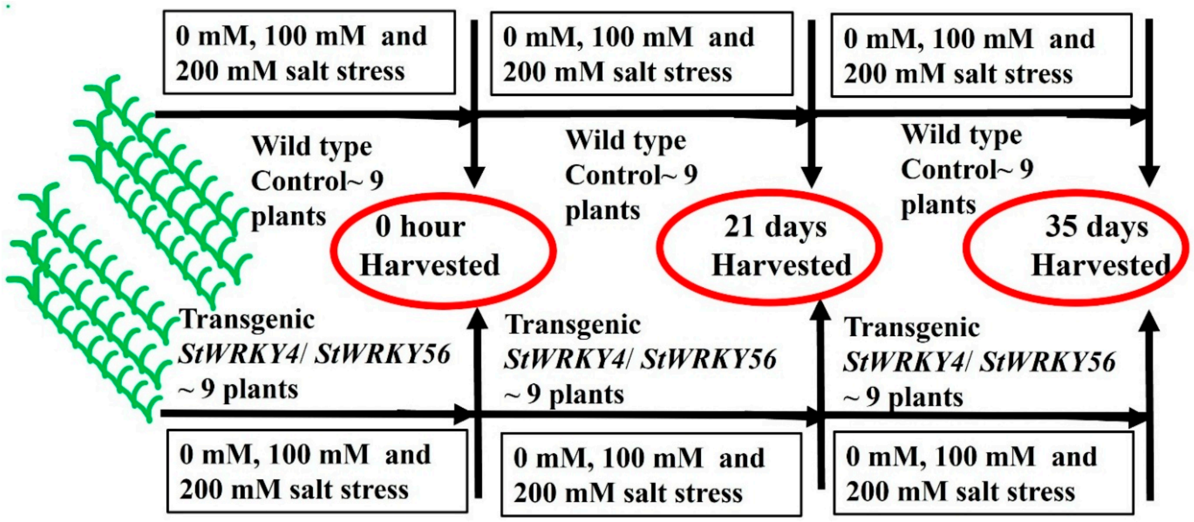

Supplementary Figure S4. Wild type, transgenic *StWRKY4* and transgenic *StWRKY56* were grown for 1 month in the growth chamber in pots containing sterilized sand and soil mix in 1:1 ratio at 25°C for 16 h photoperiod for 4 weeks and later shifted to hydroponic setup. After 35 days, ~ 27 wild type and ~ 27 transgenic plants each for *StWRKY4* and *StWRKY56* were treated with 0, 100, and 200 mm NaCl for further analysis at 0 hour, 21 and 35 days. All experiments were performed in biological triplicates.

Supplementary Table S1. Effect of salt stress on the growth, chlorophyll content, Proline, Na^+/K^+ ratio, *SOS1* and *NHX3* expressions of *StWRKY4* and *StWRKY56*. Different letters indicate a significant difference at $P \leq 0.05$ among six different treatments at three different time points according to the Duncans Multiple range test. Values are means ± SD.

Gene	Days	NaCl (mM)	No. of Leaves	No. of Roots	Shoot Length (cm)	Root Length (cm)
WRKY4	**0 days**	*St*WT	13.00 ± 1.41^{bcd}	11.50 ± 1.73^{bcd}	13.45 ± 1.14^{bc}	8.50 ± 0.55^{b}
		*St*WT 100	12.75 ± 0.96^{bcd}	12.00 ± 1.63^{bcd}	14.30 ± 1.08^{ab}	8.22 ± 0.68^{b}
		*St*WT 200	13.75 ± 0.96^{bc}	11.75 ± 0.96^{bcd}	13.30 ± 1.63^{bc}	8.15 ± 0.44^{bc}
		StWRKY4	11.75 ± 1.71^{d}	12.00 ± 0.82^{bcd}	12.55 ± 1.46^{cd}	7.53 ± 0.40^{cd}
		StWRKY4 100	12.75 ± 0.96^{bcd}	12.25 ± 0.96^{bc}	14.50 ± 1.13^{ab}	7.15 ± 0.34^{de}
		StWRKY4 200	12.50 ± 1.91^{cd}	11.00 ± 0.82^{cd}	14.88 ± 0.68^{ab}	6.86 ± 0.30^{ef}
	**21 days**	*St*WT	14.50 ± 1.29^{ab}	13.50 ± 1.73^{b}	14.68 ± 0.90^{ab}	9.15 ± 0.21^{a}
		*St*WT 100	9.50 ± 0.58^{e}	8.75 ± 1.26^{ef}	10.72 ± 1.07^{e}	6.10 ± 0.24^{g}
		*St*WT 200	9.00 ± 1.41^{e}	8.00 ± 1.41^{f}	8.90 ± 0.65^{f}	6.38 ± 0.51^{fg}
		StWRKY4	12.75 ± 0.96^{bcd}	12.50 ± 0.58^{bc}	13.43 ± 1.36^{bc}	8.10 ± 0.28^{bc}
		StWRKY4 100	9.25 ± 1.26^{e}	10.00 ± 0.82^{de}	11.45 ± 1.13^{de}	5.45 ± 0.55^{h}
		StWRKY4 200	9.75 ± 0.96^{e}	7.00 ± 1.63^{f}	10.57 ± 1.11^{e}	4.72 ± 0.61^{i}
	**35 days**	*St*WT	15.75 ± 1.26^{a}	15.50 ± 1.73^{a}	15.65 ± 1.02^{a}	9.50 ± 0.42^{a}
		*St*WT 100	5.25 ± 1.26^{f}	3.75 ± 0.50^{gh}	4.88 ± 1.03^{g}	2.98 ± 0.62^{j}
		*St*WT 200	5.00 ± 0.82^{f}	5.00 ± 1.63^{g}	3.90 ± 0.84^{g}	2.08 ± 0.28^{k}
		StWRKY4	13.50 ± 0.58^{bcd}	12.75 ± 0.96^{bc}	14.10 ± 0.64^{abc}	8.38 ± 0.22^{b}
		StWRKY4 100	4.25 ± 0.96^{f}	8.75 ± 1.26^{ef}	5.50 ± 0.58^{g}	1.88 ± 0.25^{k}
		StWRKY4 200	3.50 ± 0.58^{f}	2.25 ± 1.26^{h}	3.98 ± 1.27^{g}	1.00 ± 0.26^{l}
WRKY56	**0 days**	*St*WT	16.75 ± 1.71^{b}	12.00 ± 0.82^{bc}	13.65 ± 0.83^{c}	8.60 ± 0.53^{cd}
		*St*WT 100	16.25 ± 2.22^{b}	12.25 ± 0.96^{bc}	15.25 ± 0.96^{abc}	7.95 ± 0.33^{e}
		*St*WT 200	16.50 ± 1.91^{b}	13.50 ± 2.52^{ab}	16.32 ± 1.36^{a}	8.28 ± 0.26^{de}
		StWRKY56	16.50 ± 1.00^{b}	12.00 ± 1.41^{bc}	13.93 ± 0.83^{bc}	7.70 ± 0.43^{ef}
		StWRKY56 100	16.75 ± 1.89^{b}	13.25 ± 1.71^{b}	14.80 ± 0.24^{abc}	7.30 ± 0.32^{f}
		StWRKY56 200	16.50 ± 1.73^{b}	13.50 ± 1.73^{ab}	14.62 ± 1.92^{abc}	7.05 ± 0.47^{f}
	**21 days**	*St*WT	19.25 ± 0.96^{a}	14.25 ± 0.50^{ab}	15.15 ± 0.60^{abc}	9.38 ± 0.24^{b}
		*St*WT 100	14.00 ± 0.82^{c}	10.25 ± 1.89^{cd}	10.57 ± 1.16^{d}	6.40 ± 0.37^{g}
		*St*WT 200	12.25 ± 0.96^{cd}	9.25 ± 2.22^{de}	9.60 ± 1.49^{de}	7.08 ± 0.35^{f}
		StWRKY56	18.00 ± 1.15^{ab}	13.00 ± 1.41^{b}	15.45 ± 1.34^{abc}	8.65 ± 0.93^{cd}
		StWRKY56 100	11.75 ± 1.50^{d}	7.75 ± 0.96^{ef}	9.03 ± 2.16^{def}	6.28 ± 0.33^{g}
		StWRKY56 200	11.00 ± 0.00^{d}	8.33 ± 2.52^{def}	8.30 ± 1.13^{efg}	5.20 ± 0.30^{h}
	**35 days**	*St*WT	19.75 ± 1.26^{a}	15.75 ± 2.06^{a}	15.88 ± 0.56^{ab}	10.07 ± 0.64^{a}
		*St*WT 100	8.75 ± 0.96^{e}	8.00 ± 0.82^{def}	8.32 ± 1.77^{efg}	3.30 ± 0.22^{i}
		*St*WT 200	7.25 ± 0.96^{ef}	7.25 ± 1.26^{ef}	7.60 ± 1.20^{fg}	2.67 ± 0.40^{j}
		StWRKY56	19.25 ± 1.50^{a}	14.00 ± 1.41^{ab}	16.15 ± 1.28^{a}	9.15 ± 0.17^{bc}
		StWRKY56 100	7.50 ± 1.29^{ef}	4.50 ± 0.58^{g}	6.88 ± 0.88^{gh}	2.65 ± 0.31^{j}
		StWRKY56 200	6.00 ± 0.82^{f}	6.25 ± 0.96^{fg}	5.28 ± 0.66^{h}	2.30 ± 0.34^{j}

Gene	Days	NaCl (mM)	Chlorophyll-a (mg/g)	Chlorophyll-b (mg/g)	Carotenoid (mg/g)	Total Chlorophyll (mg/g)
WRKY 4	**0 days**	*St*WT	17.29 ± 0.52^{de}	15.53 ± 0.84^{bc}	0.57 ± 0.11^{f}	32.83 ± 0.83^{d}
		*St*WT 100	21.59 ± 1.04^{c}	16.52 ± 1.27^{ab}	0.55 ± 0.08^{f}	38.11 ± 2.21^{bc}
		*St*WT 200	24.12 ± 0.12^{ab}	14.58 ± 0.52^{cd}	0.65 ± 0.07^{ef}	38.70 ± 0.45^{b}
		StWRKY4	18.71 ± 1.29^{d}	12.39 ± 1.31^{e}	0.58 ± 0.15^{f}	31.10 ± 1.95^{de}
		StWRKY4 100	22.38 ± 0.73^{bc}	13.60 ± 0.68^{de}	0.63 ± 0.09^{ef}	35.98 ± 1.07^{c}
		StWRKY4 200	25.10 ± 1.02^{a}	16.93 ± 0.90^{a}	0.67 ± 0.08^{ef}	42.03 ± 0.68^{a}
	**21 days**	*St*WT	17.01 ± 2.87^{de}	14.86 ± 0.27^{cd}	0.67 ± 0.08^{ef}	31.87 ± 2.60^{de}
		*St*WT 100	11.87 ± 0.62^{g}	10.99 ± 0.49^{f}	0.96 ± 0.05^{bcd}	22.86 ± 0.81^{g}
		*St*WT 200	14.03 ± 0.76^{f}	6.45 ± 0.76^{h}	1.03 ± 0.05^{bc}	20.48 ± 0.86^{h}
		StWRKY4	17.97 ± 2.20^{d}	12.41 ± 1.36^{ef}	0.69 ± 0.11^{def}	30.38 ± 1.01^{de}
		StWRKY4 100	16.49 ± 1.13^{de}	8.60 ± 0.57^{g}	0.89 ± 0.10^{cde}	25.09 ± 0.56^{f}
		StWRKY4 200	15.30 ± 1.40^{ef}	7.15 ± 0.50^{h}	0.79 ± 0.10^{cdef}	22.46 ± 1.33^{gh}
	**35 days**	*St*WT	17.19 ± 1.16^{de}	14.77 ± 0.57^{cd}	0.81 ± 0.10^{cdef}	31.96 ± 1.59^{de}
		*St*WT 100	3.90 ± 0.58^{h}	4.83 ± 0.01^{i}	1.22 ± 0.36^{ab}	8.73 ± 0.59^{i}
		*St*WT 200	4.94 ± 1.56^{h}	2.05 ± 0.15^{jk}	1.31 ± 0.29^{a}	6.99 ± 1.54^{ij}
		StWRKY4	17.97 ± 0.67^{d}	12.23 ± 1.21^{ef}	0.82 ± 0.13^{cdef}	30.20 ± 1.73^{e}
		StWRKY4 100	6.02 ± 1.09^{h}	3.21 ± 0.58^{j}	1.07 ± 0.20^{abc}	9.23 ± 1.13^{i}
		StWRKY4 200	4.33 ± 0.46^{h}	1.79 ± 0.61^{k}	0.89 ± 0.06^{cde}	6.13 ± 0.28^{j}
WRKY 56	**0 days**	*St*WT	31.29 ± 2.04^{b}	12.71 ± 0.55^{c}	0.36 ± 0.09^{ef}	44.01 ± 2.58^{ef}
		*St*WT 100	32.05 ± 0.56^{b}	11.02 ± 1.75^{d}	0.39 ± 0.06^{ef}	43.07 ± 2.21^{ef}
		*St*WT 200	34.46 ± 0.59^{a}	12.01 ± 0.77^{cd}	0.41 ± 0.06^{ef}	46.47 ± 0.39^{ef}
		StWRKY56	31.23 ± 0.60^{b}	12.50 ± 0.63^{cd}	0.37 ± 0.11^{ef}	43.73 ± 1.09^{ef}
		StWRKY56 100	31.46 ± 1.33^{b}	14.20 ± 0.63^{a}	0.28 ± 0.08^{f}	45.66 ± 1.70^{f}
		StWRKY56 200	34.41 ± 2.03^{a}	14.07 ± 0.59^{ab}	0.35 ± 0.09^{ef}	48.47 ± 2.58^{ef}
	**21 days**	*St*WT	31.44 ± 1.03^{b}	12.55 ± 0.62^{c}	0.37 ± 0.09^{ef}	43.99 ± 0.95^{ef}
		*St*WT 100	20.86 ± 0.97^{d}	6.05 ± 0.83^{fg}	0.63 ± 0.06^{bcd}	26.91 ± 0.60^{bcd}
		*St*WT 200	22.86 ± 1.19^{c}	7.13 ± 0.76^{f}	0.45 ± 0.04^{def}	29.99 ± 1.63^{def}
		StWRKY56	30.89 ± 0.55^{b}	12.49 ± 0.54^{c}	0.37 ± 0.03^{ef}	43.38 ± 1.06^{ef}
		StWRKY56 100	17.24 ± 0.35^{e}	8.57 ± 0.54^{e}	0.61 ± 0.06^{cd}	25.81 ± 0.88^{cd}
		StWRKY56 200	20.35 ± 1.18^{d}	9.26 ± 0.53^{e}	0.52 ± 0.06^{de}	29.61 ± 1.11^{de}
	**35 days**	*St*WT	30.27 ± 1.11^{b}	12.65 ± 0.58^{bc}	0.37 ± 0.08^{ef}	42.92 ± 0.92^{ef}
		*St*WT 100	4.69 ± 0.63^{h}	4.03 ± 0.25^{hi}	0.76 ± 0.06^{bc}	8.72 ± 0.88^{bc}
		*St*WT 200	6.95 ± 0.91^{fg}	3.22 ± 0.24^{i}	0.96 ± 0.37^{a}	10.17 ± 0.68^{a}
		StWRKY56	30.61 ± 0.62^{b}	12.39 ± 0.72^{cd}	0.38 ± 0.03^{ef}	43.00 ± 1.34^{ef}
		StWRKY56 100	6.01 ± 0.70^{gh}	5.21 ± 1.51^{gh}	0.72 ± 0.06^{bc}	11.22 ± 2.09^{bc}
		StWRKY56 200	8.53 ± 0.52^{f}	4.05 ± 0.99^{hi}	0.82 ± 0.05^{ab}	12.58 ± 0.78^{ab}

Gene	Days	NaCl (mM)	Proline (mg/g)	Na^+ (mg/g)	K^+ (mg/g)	Na^{+}/K^{+} ratio
WRKY 4	**0 days**	*St*WT	0.25 ± 0.00[f]	3.60 ± 0.07[h]	4.40 ± 0.55[b]	0.82 ± 0.09[g]
		*St*WT 100	0.25 ± 0.01[f]	3.76 ± 0.06[gh]	4.67 ± 0.58[ab]	0.81 ± 0.10[g]
		*St*WT 200	0.25 ± 0.00[f]	3.81 ± 0.12[gh]	4.38 ± 0.00[bc]	0.87 ± 0.03[g]
		StWRKY4	0.26 ± 0.00[f]	3.78 ± 0.03[gh]	5.09 ± 0.01[a]	0.74 ± 0.01[g]
		StWRKY4 100	0.23 ± 0.00[f]	4.15 ± 0.46[fgh]	4.81 ± 0.59[ab]	0.87 ± 0.17[g]
		StWRKY4 200	0.27 ± 0.02[f]	4.48 ± 0.49[fg]	4.46 ± 0.01[b]	1.01 ± 0.11[g]
	**21 days**	*St*WT	0.28 ± 0.00[f]	3.83 ± 0.05[gh]	3.92 ± 0.01[cd]	0.98 ± 0.01[g]
		*St*WT 100	0.41 ± 0.09[e]	4.66 ± 0.55[f]	3.41 ± 0.58[ef]	1.41 ± 0.37[fg]
		*St*WT 200	0.51 ± 0.10[de]	5.54 ± 0.16[e]	2.02 ± 0.00[h]	2.74 ± 0.08[e]
		StWRKY4	0.26 ± 0.00[f]	3.85 ± 0.04[gh]	3.82 ± 0.11[de]	1.01 ± 0.02[g]
		StWRKY4 100	0.49 ± 0.01[e]	5.90 ± 0.03[de]	3.18 ± 0.15[fg]	1.86 ± 0.10[f]
		StWRKY4 200	0.62 ± 0.05[cd]	7.22 ± 1.14[c]	2.05 ± 0.01[h]	3.52 ± 0.57[d]
	**35 days**	*St*WT	0.28 ± 0.00[f]	3.86 ± 0.09[gh]	2.79 ± 0.00[g]	1.39 ± 0.03[fg]
		*St*WT 100	0.64 ± 0.10[c]	6.54 ± 0.49[d]	1.76 ± 0.01[h]	3.71 ± 0.29[d]
		*St*WT 200	0.77 ± 0.11[b]	7.52 ± 0.67[c]	1.11 ± 0.01[i]	6.79 ± 0.58[b]
		StWRKY4	0.26 ± 0.00[f]	3.95 ± 0.05[fgh]	1.96 ± 0.00[h]	2.02 ± 0.02[f]
		StWRKY4 100	0.79 ± 0.10[b]	8.80 ± 0.08[b]	1.55 ± 0.10[hi]	5.69 ± 0.38[c]
		StWRKY4 200	1.13 ± 0.16[a]	10.10 ± 0.36[a]	0.31 ± 0.00[j]	32.32 ± 1.21[a]
WRKY 56	**0 days**	*St*WT	0.13 ± 0.00[h]	0.88 ± 0.01[fg]	0.69 ± 0.01[h]	1.29 ± 0.02[efghi]
		*St*WT 100	0.18 ± 0.00[gh]	1.08 ± 0.01[efg]	1.24 ± 0.00[b]	0.87 ± 0.01[ghi]
		*St*WT 200	0.23 ± 0.05[f]	1.12 ± 0.01[ef]	1.55 ± 0.01[a]	0.72 ± 0.01[hi]
		StWRKY56	0.14 ± 0.00[h]	0.55 ± 0.07[g]	0.53 ± 0.08[i]	1.03 ± 0.13[fghi]
		StWRKY56 100	0.16 ± 0.01[gh]	0.65 ± 0.04[fg]	0.99 ± 0.01[de]	0.66 ± 0.03[i]
		StWRKY56 200	0.19 ± 0.00[fg]	0.83 ± 0.06[fg]	1.12 ± 0.06[c]	0.74 ± 0.05[hi]
	**21 days**	*St*WT	0.13 ± 0.00[h]	0.99 ± 0.00[efg]	0.65 ± 0.01[h]	1.52 ± 0.02[defghi]
		*St*WT 100	0.32 ± 0.07[e]	1.88 ± 0.01[d]	1.02 ± 0.00[d]	1.85 ± 0.01[def]
		*St*WT 200	0.47 ± 0.00[cd]	2.51 ± 0.60[c]	1.16 ± 0.01[c]	2.16 ± 0.51[d]
		StWRKY56	0.15 ± 0.01[gh]	0.65 ± 0.06[fg]	0.55 ± 0.01[i]	1.18 ± 0.11[fghi]
		StWRKY56 100	0.31 ± 0.01[e]	1.06 ± 0.01[efg]	0.52 ± 0.00[i]	2.07 ± 0.01[de]
		StWRKY56 200	0.35 ± 0.01[e]	1.47 ± 0.10[de]	0.89 ± 0.01[f]	1.65 ± 0.13[defg]
	**35 days**	*St*WT	0.14 ± 0.00[h]	1.00 ± 0.00[efg]	0.64 ± 0.01[h]	1.57 ± 0.02[defgh]
		*St*WT 100	0.43 ± 0.04[d]	3.17 ± 0.52[b]	0.76 ± 0.06[g]	4.19 ± 0.56[c]
		*St*WT 200	0.68 ± 0.00[a]	3.96 ± 0.41[a]	0.94 ± 0.01[ef]	4.21 ± 0.48[c]
		StWRKY56	0.16 ± 0.01[gh]	0.66 ± 0.04[fg]	0.53 ± 0.01[i]	1.25 ± 0.07[efghi]
		StWRKY56 100	0.48 ± 0.01[c]	2.48 ± 0.62[c]	0.29 ± 0.04[j]	8.54 ± 0.90[a]
		StWRKY56 200	0.54 ± 0.06[b]	2.93 ± 0.57[bc]	0.53 ± 0.05[i]	5.58 ± 1.48[b]

Gene	Days	NaCl (mM)	*StSOS1* Fold Change ($2^{-\Delta\Delta Ct}$)	*StNHX3* Fold Change ($2^{-\Delta\Delta Ct}$)
WRKY 4	**0 days**	*St*WT	1.00 ± 0.00[g]	1.00 ± 0.00[e]
		*St*WT 100	1.37 ± 0.05[g]	1.43 ± 0.03[e]
		*St*WT 200	1.42 ± 0.01[g]	1.25 ± 0.04[e]
		StWRKY4	1.00 ± 0.00[g]	1.00 ± 0.00[e]
		StWRKY4 100	1.37 ± 0.16[g]	1.21 ± 0.03[e]
		StWRKY4 200	1.36 ± 0.17[g]	1.14 ± 0.00[e]
	**21 days**	*St*WT	1.00 ± 0.00[g]	1.00 ± 0.00[e]
		*St*WT 100	5.71 ± 0.39[e]	5.35 ± 0.15[d]
		*St*WT 200	4.81 ± 0.50[f]	4.73 ± 0.02[d]
		StWRKY4	1.00 ± 0.00[g]	1.00 ± 0.00[e]
		StWRKY4 100	7.80 ± 0.89[d]	5.03 ± 1.36[d]
		StWRKY4 200	6.47 ± 0.07[e]	4.76 ± 0.06[d]
	**35 days**	*St*WT	1.00 ± 0.00[g]	1.00 ± 0.00[e]
		*St*WT 100	12.29 ± 0.94[b]	9.31 ± 0.56[b]
		*St*WT 200	10.81 ± 0.90[c]	12.03 ± 0.68[a]
		StWRKY4	1.00 ± 0.00[g]	1.00 ± 0.00[e]
		StWRKY4 100	15.35 ± 0.23[a]	7.51 ± 0.45[c]
		StWRKY4 200	12.51 ± 0.12[b]	6.97 ± 1.19[c]
WRKY 56	**0 days**	*St*WT	1.00 ± 0.00[f]	1.00 ± 0.00[h]
		*St*WT 100	0.84 ± 0.10[f]	0.82 ± 0.07[h]
		*St*WT 200	0.82 ± 0.10[f]	0.92 ± 0.08[h]
		StWRKY56	1.00 ± 0.00[f]	1.00 ± 0.00[h]
		StWRKY56 100	0.98 ± 0.01[f]	0.76 ± 0.01[h]
		StWRKY56 200	0.82 ± 0.00[f]	0.79 ± 0.01[h]
	**21 days**	*St*WT	1.00 ± 0.00[f]	1.00 ± 0.00[h]
		*St*WT 100	6.09 ± 1.17[e]	3.91 ± 0.11[e]
		*St*WT 200	5.17 ± 1.02[e]	3.05 ± 0.17[f]
		StWRKY56	1.00 ± 0.00[f]	1.00 ± 0.00[h]
		StWRKY56 100	5.48 ± 0.72[e]	2.93 ± 0.04[f]
		StWRKY56 200	6.32 ± 0.70[e]	2.23 ± 0.20[g]
	**35 days**	*St*WT	1.00 ± 0.00[f]	1.00 ± 0.00[h]
		*St*WT 100	13.00 ± 0.76[c]	6.24 ± 0.06[b]
		*St*WT 200	11.51 ± 0.19[d]	8.15 ± 0.90[a]
		StWRKY56	1.00 ± 0.00[f]	1.00 ± 0.00[h]
		StWRKY56 100	14.95 ± 0.67[b]	5.56 ± 0.55[c]
		StWRKY56 200	16.56 ± 0.65[a]	4.96 ± 0.06[d]
